# Peripheral Nerves in Cancer: Regulatory Roles and Therapeutic Strategies

**DOI:** 10.1002/mco2.70594

**Published:** 2026-01-18

**Authors:** Yan Fu, Zhi‐Shan Ge, Qing‐Yue Cao, Zi‐Han Li, An Zhang, Hai‐Dong Zhu, Gao‐Jun Teng

**Affiliations:** ^1^ Center of Interventional Radiology & Vascular Surgery, Nurturing Center of Jiangsu Province For State Laboratory of AI Imaging & Interventional Radiology (Southeast University), Department of Radiology, Zhongda Hospital, Medical School Southeast University Nanjing China; ^2^ State Key Laboratory of Digital Medical Engineering, National Innovation Platform for Integration of Medical Engineering Education (NMEE) (Southeast University), Basic Medicine Research and Innovation Center of Ministry of Education, Zhongda Hospital Southeast University Nanjing China

**Keywords:** cancer, interaction, mechanism, nerve, therapeutic strategies

## Abstract

Cancer neuroscience has emerged as a transformative frontier in oncology research, focusing on the interplay between cancer cells and the nervous system. Cancer cells establish tumorspecific neural networks within tumor tissues via neurotrophic hijacking. The nervous system regulates tumor initiation, progression, and metastasis either directly by regulating signal transduction in tumor cells or indirectly by modulating the tumor microenvironment (TME). The positive feedback loop between cancer cells and nerves promotes tumor progression. Deciphering the regulatory role of nerves in tumor progression may yield novel anticancer therapeutic options. In this review, the interaction between nerves and cancer cells is described, including how cancer cells hijack and remodel nervous system structure and function, and how neuron‐signaling regulates cancer cell growth directly or indirectly through modulating the TME. This evidence of the critical role of nerves in the malignant phenotype of tumors indicates the potential of using neuron‐signaling targeting strategies in cancer treatment. By summarizing these findings, this review aims to provide comprehensive insights into the interaction between nerves and cancer cells, paving the way for neuron‐signaling‐based anticancer therapies.

## Graphical abstract

The interaction between cancer cells and peripheral nerves: (1) tumor cells could induce the growth of new neural axons and constructing a neural fiber network within the tumor tissue through secreting nerve growth factors; (2) nerve‐derived bioactive molecules can induce perineural invasion of tumor and regulate the malignant phenotype of tumor, such as immune invasion, proliferation, metastasis, angiogenesis, and therapy resistance (created with BioRender.com).

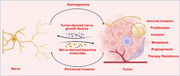

## 1 Introduction

Cancer remains one of the most formidable challenges to global health, with more than 19 million diagnoses and 9 million deaths worldwide each year [1]. Cancer is not an autonomous entity but a complex ecosystem composed of tumor cells and their environment. The tumor microenvironment (TME), composed of various types of cellular and noncellular constituents, including immune cells, fibroblasts, endothelial cells, extracellular matrix (ECM), and hypoxia, plays a significant role in regulating the malignant phenotype of the tumor, including tumor initiation, invasion, metastasis, and therapy resistance [2]. A growing body of evidence has highlighted a critical yet historically understudied player in this ecosystem, the nervous system. The nervous system, which plays a central role in the physiological functions of all organs, has emerged as a critical regulator of tumor progression. Perineural invasion (PNI), a phenomenon characterized by tumor cells surrounding and spreading along nerve fibers, represents the earliest understanding of the interaction between nerves and tumor cells [3, 4]. PNI is a significant indicator of the malignant phenotype of tumor and correlates with the poor prognosis of patients [4, 5]. Recently, numerous studies have revealed the regulatory role of nerves in tumor initiation, proliferation, and metastasis. Pioneering work by Magnon et al. revealed that prostate cancer is infiltrated by autonomic nerves, including adrenergic nerves which play a significant role in the early stage of prostate cancer development, and the cholinergic nerves, which contribute to tumor dissemination in later stages [6]. Numerous studies have since reported the crucial role of the interplay between cancer cells and nerves in regulating tumor progression. The interaction between peripheral nerves and cancer cells, as well as the critical regulatory role of this interaction in tumor progression, have been documented in several other cancers, including gastric cancer [7], pancreatic cancer [8], breast cancer [9], colon cancer [10], head and neck cancer [11], and cholangiocarcinoma [12]. Targeted inhibition of the interaction between cancer cells and peripheral nerves could potentially inhibit tumor progression [13]. These studies represent the emergence of a new field in cancer research, known as cancer neuroscience [14].

**FIGURE 1:**
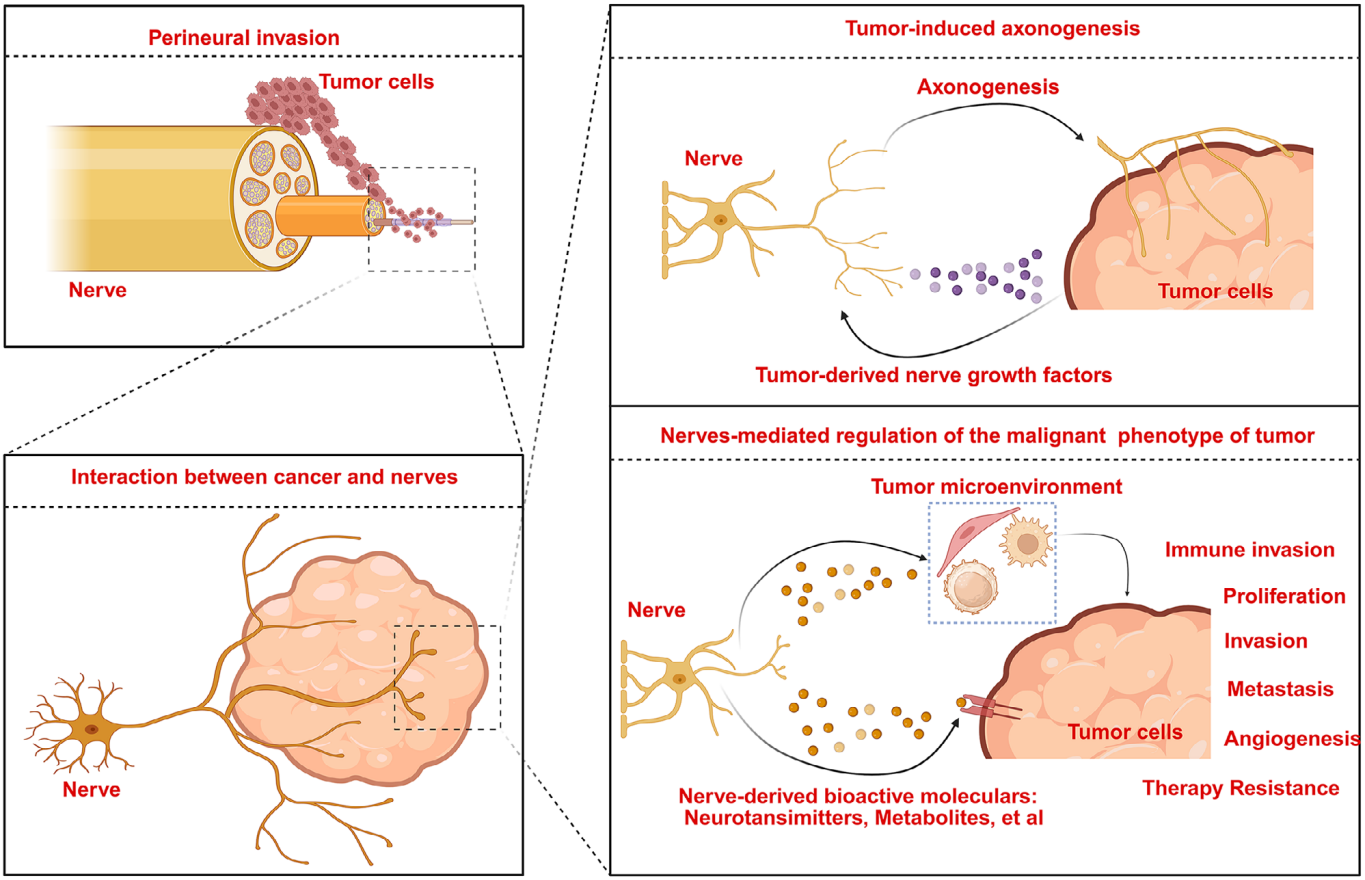
The interaction between cancer cells and nerves. Tumor cells can induce the growth of new neural axons and form a neural fiber network within the tumor tissue by secreting nerve growth factors, including NGF, BDNF, and GDNF. The infiltrated nerves could regulate the malignant phenotype of the tumor by releasing numerous neurotransmitters, metabolites, and growth factors. (This figure was created using BioRender.com.)

Cancer neuroscience, which focuses on deciphering the interactions between nerves and cancer cells, has provided new insights into the mechanisms of cancer initiation and progression. The relationship between peripheral nerves and tumors can be summarized as follows (Figure 1): (1) tumor cells can induce the growth of new neural axons and construct neural fiber networks within the tumor tissue by secreting nerve growth factors (NGFs), such as NGF, brain‐derived neurotrophic factor (BDNF), and glial cell line‐derived neurotrophic factor (GDNF); (2) infiltrated nerves can in turn, regulate the malignant phenotype of tumor via releasing a variety of neurotransmitters, metabolites, and growth factors; (3) infiltrated nerves can also stimulate cancer cells to secrete neurotrophic factors to facilitate the outgrowth of nerves, forming a positive feedback loop between cancer cells and nerves to promote tumor progression; (4) nerves can release neurotransmitters or signal transduction factors to modulate the TME, particularly the immune microenvironment, thereby exerting an indirect effect on tumor progression. Considering the crucial role of nerves in tumor progression, targeting the dysregulated interactions between nerves and cancer cells may represent a novel therapeutic strategy for cancer treatment. Therefore, uncovering the underlying mechanisms by which nerves regulate tumor progression could provide valuable insights into new anticancer therapeutic strategies.

In this review, the interaction between nerves and tumors as well as its role in cancer progression is comprehensively described. First, we delineated the evolution of the concept regarding the interaction between cancer and nerves, from the traditional understanding of PNI to the current recognition of mutual regulation between nerves and tumors. Subsequently, we provided an overview of the mechanisms by which cancer cells induce neurogenesis and how nerves stimulate the malignant phenotype of tumors, either directly by promoting signal transduction in tumor cells or indirectly by modulating the TME. We have discussed the potential therapeutic applications of neural signaling‐based anticancer treatments. Through this structured review, we illustrated the interactions between nerves and cancer cells, their roles in cancer progression, and the underlying regulatory mechanisms, with the hope of paving the way for developing cancer neuroscience‐based anticancer strategies.

## 2 PNI: A Traditional Perspective on the Relationship Between Cancer and Nerves

The description of PNI, an initial understanding of the interaction between nerves and tumors, was conducted over a century ago. PNI is one of the key tumor invasion mechanisms, characterized by tumor cells infiltrating at least 33% of the nerve circumference or invading the nerve sheath [15]. It has been identified as an independent adverse prognostic factor in cancers [16]. The earliest studies suggested that PNI occurs because tumor cells passively disseminate through the loose perineural space between the nerve and its sheath with weak resistance [3, 17]. However, later studies revealed that the nerve sheath, composed of multiple layers of collagen, is highly resistant [18], suggesting that PNI might be a result of aggressive invasion rather than passive dissemination. Recent evidence indicates that specific molecular signals, mediated by neurotrophic factors, chemokines, and axon guidance molecules in the perineural niche, stimulate cancer cell invasion along nerves [19, 20, 21, 22, 23, 24]. PNI is a complex process that includes several stages: (1) Synthesis of adhesion‐inhibiting molecules to help cancer cells detach from the primary tumor mass; (2) secretion of ECM‐degrading proteolytic enzymes, which facilitate tumor cell invasion into the nerve sheath; (3) induction of nerve stimulation symptoms or dysfunction of the innervated nerves; (4) tumor growth and spread within the nerve sheath. PNI is a dynamic, bidirectional crosstalk between cancer cells and nerves, involving reciprocal signaling mechanisms rather than passive cancer cell infiltration of neural structures. This bidirectional cellular crosstalk regulates both neurotropic attraction and tumor progression.

### 2.1 The Role of Tumor Invasion Ability in PNI

Cancer cells actively participate in the PNI. Adhesion molecules play pivotal roles in cancer cell migration along nerve fibers by dynamically regulating tumor‐neural cell adhesion and remodeling the ECM to facilitate PNI and dissemination. Several adhesion molecules, including cadherins, integrins, and immunoglobulin superfamily adhesion molecules, mediate PNI in cancer. Epithelial–mesenchymal transition (EMT) is a key mechanism that promote cancer cell invasion and migration. There is a mechanistic link between EMT inducers and PNI. Tumor cells undergo downregulation or loss of E‐cadherin to disrupt intercellular adhesion, enabling the dissociation of cancer cells from primary lesions and facilitating perineural infiltration and progression [25]. Matrix metalloproteinases (MMPs) are a family of enzymes that regulate ECM remodeling. Elevated MMP expression is significantly correlated with enhanced tumor invasiveness and increased PNI incidence. These enzymes promote neural infiltration through targeted degradation of perineural matrix barriers, such as collagen fibers surrounding nerves [26]. For example, pancreatic cancer cells secrete MMP1, which activates the protease‐activated receptor 1 (PAR1) in pancreatic neurons. This triggers AKT‐mediated substance P (SP) release, subsequently activating neurokinin‐1 receptor on tumor cells to upregulate ERK1/2 signaling. The resulting enhancement of tumor cell migration, invasion, and PNI establish a self‐reinforcing MMP1–PAR1–SP signaling circuit that progressively amplifies neural infiltration [27]. Upon breaching the perineurium and entering the intraneural space, cancer cells significantly upregulate integrin molecules, enhancing their neural invasive capacity and proliferation [28]. In addition, cancer cells respond dynamically to elevated ECM stiffness by upregulating NGF expression, facilitating interactions between cancer cells and nerve structures, and ultimately promoting PNI [29].

### 2.2 The Role of Microenvironmental Cues in PNI

Beyond their autonomous invasive capacity, tumor cells respond to neural microenvironmental cues that promote PNI. The inflammatory microenvironment around the nerves, caused by cancer cell‐mediated damage to nerve fibers, plays an important role in PNI. This inflammatory microenvironment is rich in neural homeostasis‐regulating molecules, including chemokines that induce cancer cell chemotaxis toward nerves and promote their survival and proliferation [30, 31, 32]. C‐C motif chemokine ligand 2 (CCL2) is a nerve‐secreted chemokine that mediates the neural invasion of CCR2‐positive cancer cells [32]. Moreover, CCL2 can attract CCR2‐positive macrophages to the immune microenvironment of neuritis [32], where they promote neural invasion by activating RET signaling in cancer cells [33, 34]. CXCL12, a chemokine secreted by nerves, mediates the chemoattraction of CXCR4‐positive cancer cells, facilitating their migration toward nerves via a concentration gradient [35]. Marchesi et al. reported that neurons can secrete CX3CL1, which binds to CX3CR1 on tumor cells, enhancing tumor cell invasion and inducing cancer cell chemotaxis toward nerves, thereby promoting PNI [31]. In addition to chemokines, neurotrophins, including NGF, GDNF, and neuregulin‐1, play significant roles in promoting PNI. NGF, primarily secreted by neurons and glial cells, is a well‐studied neurotrophic factor that plays a critical role in regulating neuronal growth [36]. Multiple studies have demonstrated the regulatory role of NGF in PNI [37]. NGF can bind to its receptor tropomyosin receptor kinase A (TrkA), inducing directional invasion of tumor cells toward nerves [38]. Targeting the NGF–TrkA axis can diminish cancer cell migration along neurites in vitro and inhibit PNI in vivo [39]. GDNF, a neurotrophic factor secreted by damaged nerves or glial cells, can activate the GFRα cognate tyrosine kinase receptor, thereby stimulating the directional migration and invasion of cancer cells [40, 41]. Chen et al. reported that Schwann cell‐derived GDNF triggers MUC21 phosphorylation, facilitating its interaction with RAC2. This cascade causes RAC2 membrane localization and activation, inducing EMT to promote cancer metastasis and PNI [42]. In addition to NGF and GDNF, BDNF increases cancer cell invasion and chemotaxis toward nerves through TrkB‐mediated activation of phosphoinositide 3‐kinase (PI3K)/AKT and mitogen‐activated protein kinase (MAPK)/ERK signaling pathways [43].

Neurotransmitters are important nerve‐derived signaling molecules. Recent studies have demonstrated that PNI can be stimulated by neurotransmitters secreted from nerves [44, 45, 46]. The sympathetic neurotransmitter noradrenaline enhances neural invasion along dorsal root ganglion (DRG) neurites through β‐adrenergic receptor/signal transducer and activator of transcription 3 (STAT3) signaling‐mediated activation and upregulation of MMPs in cancer cells [47]. Our previous study demonstrated that the acetylcholine (Ach)/CHRNA5 axis promotes PNI progression in cholangiocarcinoma by upregulating BDNF expression through activation of the CAMKII/GSK3β/β‐catenin signaling pathway [12].

Several other cellular components within the TME are involved in regulating PNI. Cancer‐associated fibroblasts (CAFs) are important components of TME. Zheng et al. reported that CAF‐derived extracellular vesicles (EVs) promote PNI in pancreatic ductal adenocarcinoma (PDAC). CAF‐derived EVs mediate the transfer of the PNI‐associated transcript to PDAC cells, where it binds to YBX1 to inhibit YBX1 degradation by blocking the YBX1–Nedd4l interaction. Furthermore, YBX1 binds to PNI‐associated mRNAs in a 5‐methylcytosine‐dependent manner, ultimately upregulating PNI‐associated genes, including EGR1, NTRK1, and SMAD7 [48]. Moreover, CAFs exhibit upregulated glycolysis due to GAPDH acetylation, resulting in increased lactate production. Lactate promotes histone H3K18 lactylation, increasing the transcription of neural invasion‐associated genes such as *L1CAM* and *SLIT1*, thereby driving PNI in pancreatic cancer [49]. Schwann cells are critical cellular components of the neural microenvironment that play essential roles in neural repair and regeneration [50]. Tumor‐mediated nerve damage triggers the migration of Schwann cells from undamaged nerve segments to injury sites. These activated Schwann cells can guide axons into the cancer mass, potentially facilitating tumor cell spread and modifying the local microenvironment to support tumor growth and progression [51]. Stellate cells, another key component of the nerve sheath microenvironment, are activated by cancer cell‐derived TGF‐β. This activation increases MMP transcription, promoting local cancer cell growth and invasion to facilitate PNI [50, 52, 53].

In summary, various cellular and noncellular components within the TME participate in regulating PNI progression. A better understanding of the mechanisms underlying PNI could pave the way for the identification of potential therapeutic targets for PNI.

## 3 Positive Feedback Loops Between Cancer and Nerves: The Current Cognition of the Interaction Between Cancer and Nerves

Recent studies have revealed that cancer–nerve interactions extend far beyond PNI, marking a significant paradigm shift in our understanding of tumor neurobiology. Unlike PNI, a traditional understanding of the interaction between cancer cells and nerves, the innervation of cancer, characterized by increased density of nerve fibers within tumors, suggests that nerves may be actively recruited by cancer cells. Ayala et al. elucidated this bidirectional interaction through coculture experiments with DRG and prostate cancer cells. The nerve fibers extended directionally toward tumor cells, while cancer cells migrated along neural axons toward DRG upon direct contact. These findings indicate a dynamic, reciprocal relationship between tumors and nerves [54]. Tumor innervation, characterized by increased nerve density during cancer progression from preneoplastic lesions to overt malignancy, represents a recognized histological alteration in neoplastic tissues [55]. This neural infiltration has been documented across multiple solid tumors, with a significantly higher density of nerve fibers within tumor tissues than in adjacent normal tissues [55, 56]. Nerve fibers are present in tumors even without PNI, and their increased density correlates with patient prognosis regardless of PNI status [57]. These observations raise two fundamental questions: (1) What mechanisms drive nerve fiber recruitment into tumors, and (2) how does increased nerve density correlate with clinical outcomes? These questions are addressed in the following sections.

## 4 Neurogenesis: The “Conspiracy” of Tumors

Kovacs et al. discovered that cancer cells demonstrated an increased ability to recruit nerves in response to chemotherapy and that nerves could subsequently promote tumor growth and contribute to treatment resistance [58]. These findings demonstrate that nerve infiltration in tumor tissues might be a “conspiracy” of tumor tissues, enabling them to obtain neurosecretory molecules to satisfy the demands of tumor cells [59]. Similar to angiogenesis, wherein tumor cells induce neovascularization to facilitate nutrient supply, neurogenesis can be carried out by cancer cells to induce the infiltration of nerves into the tumor mass, thereby augmenting the malignant phenotype of the tumor. Similar to the process by which tumor cells induce angiogenesis by secreting vascular endothelial growth factor (VEGF) family factors, they can also secrete neurotrophic factors, including BDNF, NGF, NTF3, and NTF4. These factors activate neurotrophic factor receptors on neural axons, inducing neurite outgrowth and creating a tumor‐specific neural network within the tumor. The nerve terminals in the TME can regenerate and extend under the influence of neurotrophic factors, significantly increasing the density of nerve fibers within the tumor tissue without apparent neural invasion.

**FIGURE 2:**
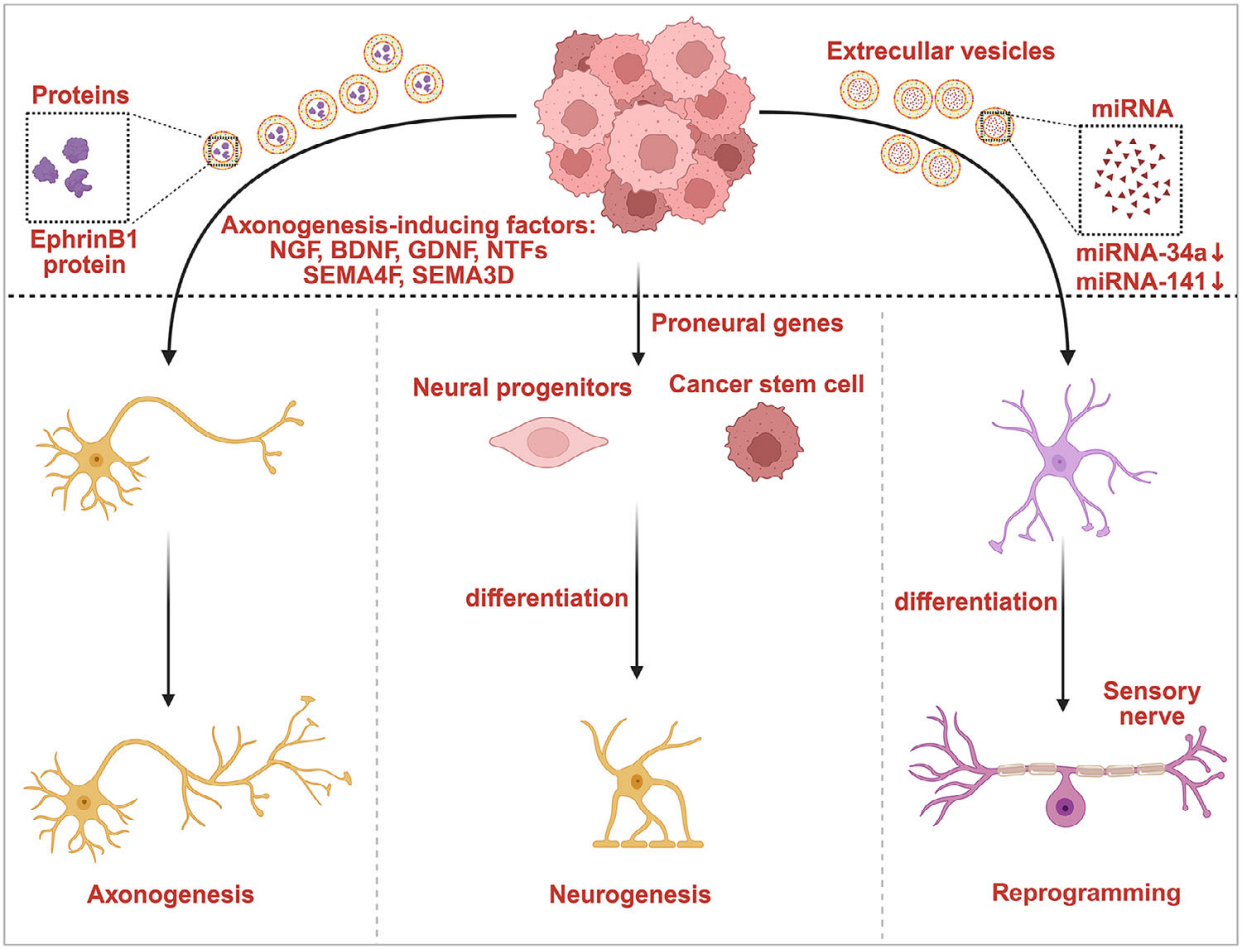
Neurogenesis in tumor. The three main sources of nerve fibers in tumors include the following: (1) Tumor cell‐induced infiltration of nerve fibers derived from the outgrowth of nerves that were originally “residing” in normal tissues, primarily by secreting axonogenesis‐inducing factors or EVs containing axonogenesis‐associated; (2) migration and reprogramming of neural progenitor cells from the remote locations toward the tumor mass; (3) differentiation of tumor stem cells into functional “neuron‐like cells.” (This figure was created using BioRender.com.)

In addition to the outgrowth of nerves originally residing in normal tissues (the main source of nerve fibers in the tumor), tumor tissues may acquire nerve fibers through two additional mechanisms (Figure 2): (1) Migration of neural progenitor cells from distant locations to the tumor mass; (2) differentiation of tumor stem cells into functional neuron‐like cells [60]. The differentiation of neural progenitor cells and tumor stem cell‐generated nerve fibers in tumors remains poorly understood. In 2019, Mauffrey et al. reported that DCX‐positive neural progenitor cells from the central nervous system (CNS) can traverse the blood–brain barrier and migrate to prostate cancer tissues through the bloodstream and reside in the tumor tissues, where they generate new adrenergic neurons that may contribute to prostate cancer progression [61]. Zhang et al. performed genomic analysis of prostate cancer stem‐like progenitor cells and discovered a neural‐like gene expression signature that correlated with the prognosis of patients with prostate cancer, implying that these cells can differentiate into neural cells and contribute to the malignant phenotype of cancer cells [62]. Similarly, Lu et al. reported that stem‐like progenitor cells in colon and gastric cancers can differentiate into neural lineages, and inhibiting this differentiation capacity can suppress the tumorigenic potential of the tumor cells [63]. However, further research is required to determine whether neural infiltration resulting from nerve fibers derived from remote neural progenitor cells or the tumor stem cells differentiation is universally applicable across different cancer types. Currently, most research on infiltrated nerves in tumor is mainly focused on the outgrowth of nerves originally “residing” in normal tissues.

### 4.1 Factors Contributing to Neurogenesis

Emerging evidence indicates that cancer cells can induce axon formation by secreting NTFs, axon guidance molecules, and EVs. NTFs, including neurotrophin‐3 (NT‐3), NGF, NT‐4, and BDNF, play crucial roles in neuronal survival, development, differentiation, and regeneration by binding to specific receptors on the neuronal surface and activating several signaling pathways. They also mediate cancer‐associated neural plasticity within the TME. The ability of cancer cells to secrete neurotrophic factors has been documented since the previous century, establishing their role in tumor–nerve crosstalk [64]. Recent studies suggest that cancer cell‐derived neurotrophic factors can induce neural axon neogenesis and promote neural infiltration by activating neurotrophic factor receptor‐based signaling in nerves.

#### 4.1.1 NGF

NGF, the first neurotrophic factor to be comprehensively described, is involved in neuronal growth, development, and injury repair by activating TrkA and p75 receptors. Clinical studies demonstrate that NGF expression is associated with cancer‐associated neuroplasticity and advanced tumor grade [9, 65]. In prostate cancer, tumor cells secrete proNGF to induce axonogenesis, thereby promoting neural infiltration [65]. This phenomenon extends to breast, gastric, and pancreatic cancers, where NGF secretion drives axonogenesis and facilitates neural network formation within tumors [9, 66, 67, 68], highlighting the crucial role of NGF in the construction of neural networks within tumors.

#### 4.1.2 BDNF

BDNF, another well‐characterized neurotrophic factor, can bind toTrkB and activate intracellular signaling pathways, including PI3K and MAPK, regulating neuronal growth and development. Recently, the regulatory role of BDNF in tumor‐associated axonogenesis and neural infiltration has been elucidated. In PDAC, tumor‐derived BDNF promotes axonogenesis and increases nerve density in the tumor mass [66]. Similarly, ovarian cancer cells upregulate BDNF expression via the ADRB3/cAMP/Epac/JNK axis in response to catecholamines, inducing axonogenesis and increasing the density of nerves in the tumor mass [69]. Moreover, we discovered that Ach signaling could stimulate BDNF expression, increasing nerve infiltration in cholangiocarcinoma [12].

#### 4.1.3 Axon Guidance Factors

The axon guidance factors, which function as chemo‐attractants and guidance cues during neural development, were also reported to play a crucial role in the process of axonogenesis in tumor. The emaphorin4F (SEMA4F), a kind of axon‐guidance factor, overexpressed in prostate cancer cells, could enhance the ability of prostate cancer cells to induce neurite outgrowth [55]. Clinical analyses demonstrated that SEMA4F expression levels significantly correlate with both intratumoral nerve density and the extent of PNI in prostate cancer [70]. Similarly, Jurcak et al. found that the axon guidance molecule SEMA3D, secreted by cancer cells, could activate the PLXND1 on the surface of neural cells, further inducing neurite outgrowth and promoting neural invasion in pancreatic cancer [71].

#### 4.1.4 EVs

EVs, nanoscale double‐layered membrane structures with diameters ranging from 30 nm to 9 µm, play crucial roles in axonogenesis in tumors. The bioactive molecules in EVs, including proteins, mRNAs, noncoding RNAs, lipids, and metabolites, are closely associated with intercellular communication [72]. Emerging evidence indicates that tumor cell‐derived EVs play important roles in neurite outgrowth and neural infiltration. Christopher et al. discovered that PC12 pheochromocytoma cells, cocultured with EVs derived from cervical cancer cells, exhibited an increased ability to generate neuronal axons, implying a potential role of EVs in guiding axonogenesis [73]. Furthermore, Marianna et al. discovered that head and neck tumor cell‐secreted EVs containing the axon guidance molecule EphrinB1 could induce axonogenesis and increase the intratumoral nerve density [11]. TP53‐mutated head and neck tumor cells generate EVs with decreased miR‐34a expression, which can promote the growth of neuronal axons and induce the differentiation of neuronal cells into a sympathetic neuronal phenotype [74]. While these findings establish the role of EVs in neural remodeling, key questions remain, including whether other components carried by EVs play a role in nerve infiltration. Current research on EV‐mediated axonogenesis and nerve infiltration is limited, warranting further investigation.

In summary, cancer cells use multiple mechanisms, including secretion of neurotrophic factors, axon guidance molecules, and EVs, to drive tumor‐associated axonogenesis. Elucidating these neural remodeling processes may reveal novel therapeutic targets for disrupting the tumor–nerve axis in cancer treatment.

## 5 Nerves Regulate the Malignant Phenotype of Cancer Cells

Although the phenomenon of nerve fibers infiltrating the TME has been observed since the early 20th century, nerve fibers were originally considered passive bystanders in tumorigenesis [75, 76]. Early histological studies demonstrated that electrical stimulation of the superior cervical ganglion, which is responsible for the expansion of adrenergic nerves in salivary glands, facilitates glandular hyperplasia [77, 78], suggesting a potential regulatory role of nerves in tumor initiation. Ayala et al. discovered that cancer cells invading nerve bundles in prostate cancer exhibited a significantly reduced rate of apoptosis and an increased rate of proliferation compared with tumor cells far from nerves. This difference may be attributed to the nerve‐mediated increased activity of nuclear factor kB and its downstream targets, PIM‐2 and DAD‐1 protein [22]. In vitro assay conducted by Dai et al. revealed that cancer cells cocultured with DRG cells exhibited significantly enhanced proliferation ability and apoptosis resistance, further highlighting the potential regulatory role of nerves in the malignant phenotype of tumors [79]. Subsequent research revealed that neurotrophic growth factors‐overexpressing tumor cells could induce nerve infiltration into the tumor mass, activating the nerve‐dependent pathways to facilitate growth of tumor during tumor initiation and progression. Magnon et al. systematically investigated the regulatory role of nerves in tumorigenesis for the first time. The result revealed that denervation treatment can significantly inhibit the occurrence and development of prostate cancer [45], catalyzing extensive research into neural regulation of tumors. Herein, the mechanisms underlying the regulatory role of nerves in tumor progression are discussed.

### 5.1 Direct Regulatory Effect of the Nerve System on Tumor Progression

**FIGURE 3:**
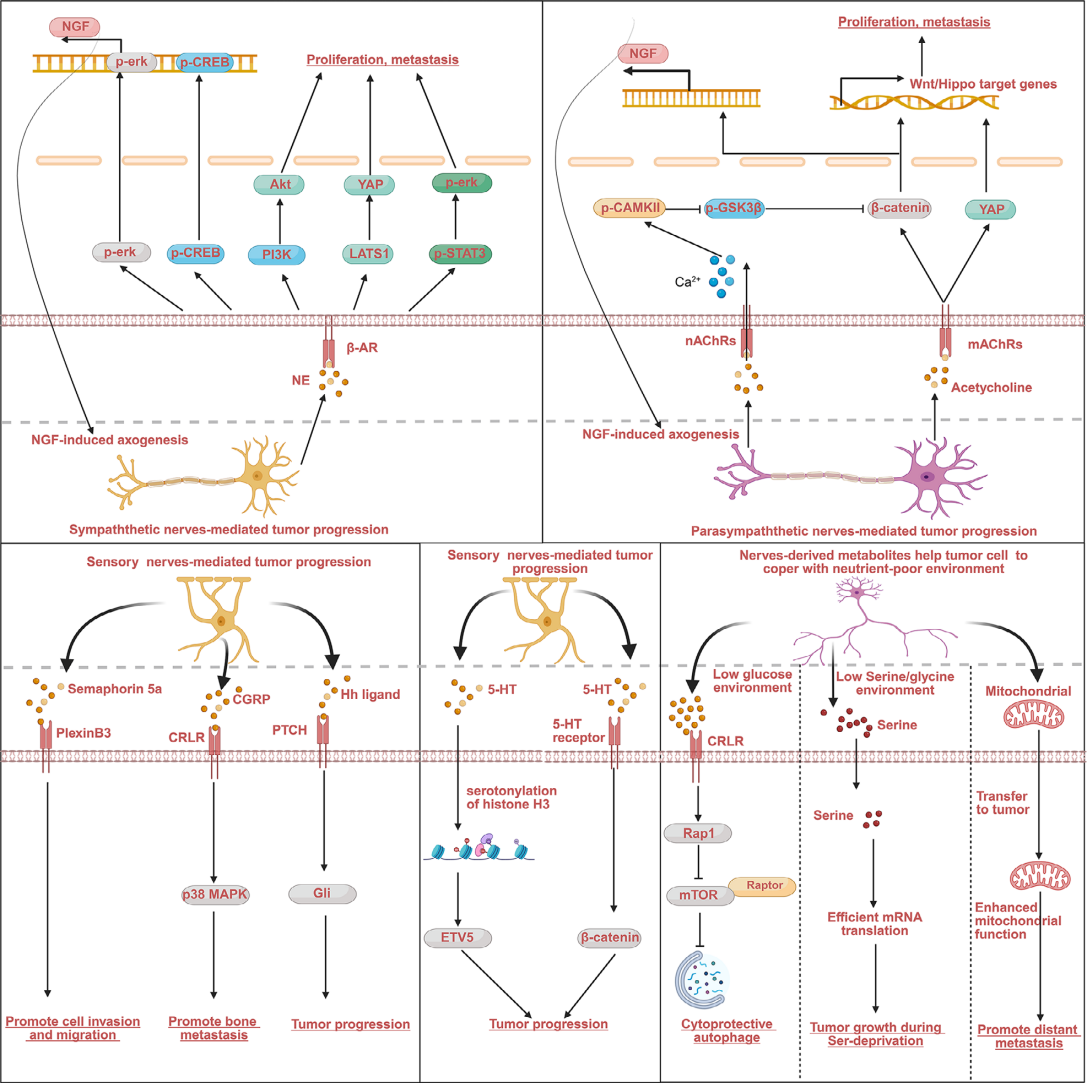
Direct regulatory effects of nerves on tumor cells. Nerves, including sympathetic, parasympathetic, and sensory nerves, could regulate the malignant phenotype of the tumor by activating corresponding receptors and their downstream signaling. Nerves can also regulate tumor metabolic plasticity, promoting tumor growth in nutrient‐poor environments. (This figure was created using BioRender.com.)

Magnon et al. demonstrated that autonomic nerve fibers in the prostate gland regulate prostate cancer development and dissemination [6]. Subsequent studies have established the involvement of peripheral nerves in the progression of numerous malignancies. During tumor initiation and progression, cancer cells engage in nerve‐dependent signaling pathways to promote growth and metastatic dissemination. The neural regulation of malignant phenotypes of tumor cells was achieved mainly through the secretion of paracrine factors. After tumors innervation, tumor cells‐derived paracrine factors, including neurotransmitters, metabolites, and soluble proteins, directly regulate the malignant phenotype of tumor cells (Figure 3).

#### 5.1.1 Neurotransmitter‐Mediated Regulatory Effect of Nerves on Tumor Progression

Neurotransmitters mediate of neural regulation of physiological processes. Neurotransmitters can be divided into three categories based on their chemical structure: amino acids (Ach, gamma‐aminobutyric acid, glycine [Gly], and glutamate), biogenic amines (epinephrine, norepinephrine, and serotonin), and peptidergic neurotransmitters (SP, neuropeptide Y, and neurotensin [NTS]) [80]. Growing evidence demonstrates that neurotransmitters derived from the central or peripheral nervous system play significant roles in cancer progression. NTS, a neuropeptide initially identified in bovine hypothalamus, is a prominent CNS‐derived neuropeptide that contributes to tumor progression through multiple oncogenic pathways [81, 82, 83]. NTS binds to its cognate receptor, NTSR1, and activates downstream oncogenic signaling pathways, such as PI3K/Akt, thereby promoting tumor progression [84]. The regulatory role of NTS/NTSR1 signaling in tumor progression has been comprehensively reviewed [85, 86]. In addition, peripheral nervous system‐derived neurotransmitters play a significant role in tumor progression, and this role will be comprehensively discussed in the following section.

Norepinephrine, the primary effector of adrenergic signaling, regulates the progression of several types of tumors by directly activating the adrenergic receptor signaling pathway [87]. Early investigations into stress‐induced tumor progression revealed a functional link between sympathetic nervous system activation and cancer biology. Chronic stress can increase the activity of the adrenergic nervous system, thereby increasing the concentrations of circulating and intratumoral catecholamines [88, 89, 90]. A recent study reported that psychological stress stimulates sympathetic nerve innervation in PDAC [91]. Sympathetic nerves‐activated adrenergic signaling induces widespread N6‐methyladenosine (m6A) modification of RNAs by downregulating RNA demethylase alkB homologue 5 in tumor cells. These m6A‐modified RNAs are selectively packed into EVs and transferred to nerves, thereby creating a feedforward loop that sustains hyperinnervation and promotes tumor progression [91]. Preclinical studies have demonstrated that chronic stress‐induced adrenergic signaling pathway activation promotes the progression of multiple cancer types, including breast, ovarian, prostate, and pancreatic cancers [89, 92, 93, 94]. Under chronic stress, resection of the bilateral adrenal glands, the primary producer of catecholamines in circulation, significantly attenuated tumor progression in the transgenic pancreatic tumor model, whereas no effect was observed in the unstressed mice [66]. Under the pressure of tumor burden, sympathetic nerves with a polysynaptic connection to the brain can infiltrate the tumor mass, where they act as transmitters of adrenergic signaling from corticotropin‐releasing hormone neurons in the central medial amygdala to stimulate tumor progression [95]. These findings demonstrate that adrenergic signaling regulates tumor malignancy through both systemic neurohormonal effects and direct peripheral nerve–tumor interactions.

There is evidence of direct interaction between peripheral sympathetic nerves and cancer cells, with norepinephrine serving as a crucial regulator of the malignant phenotype of tumors. Raju et al. discovered that bilateral sympathectomy suppresses tumor proliferation and invasion in oral squamous cell carcinoma, indicating an active role of sympathetic nerves in cancer progression [96]. In head and neck squamous cell carcinoma, sympathetic nerve‐derived norepinephrine activates β‐adrenergic receptors, upregulating tumor necrosis factor‐alpha (TNF‐α) production and driving tumor growth and nociceptive signaling [97]. Magnon et al. reported that sympathetic nerves‐derived norepinephrine activates the β2‐ and β3‐adrenergic receptors, driving tumor initiation and progression in the early phase of prostate cancer development [6]. Clinical observations demonstrate that β‐blocker therapy delays progression to castration‐resistant prostate cancer (CRPC), and preclinical studies further confirmed that β‐blocker therapy reduced tumor burden and significantly postponed CRPC development [98]. β‐blockers suppress androgen receptor (AR) signaling, leading to inhibition of the PI3K/AKT/mTOR pathway and a metabolic reprogramming from fatty acid synthesis to oxidative phosphorylation [98]. In triple‐negative breast cancer (TNBC), sympathetic nerves are closely associated with the malignant progression of the tumor [99], as well as the poorer prognosis of patients with TNBC [100]. Zuo et al. further confirmed that sympathetic denervation could improve the anticancer efficacy of chemotherapy and relieve chemotherapy‐induced neuropathic pain in breast cancer [100]. In pancreatic tumors, cancer cells secrete NGF to recruit nerve fibers into TME. The infiltrated sympathetic nerves activate β2 adrenergic s/p‐CREB/p‐Erk signaling to promote tumor progression [66]. Stomach adenocarcinoma tissues exhibit significantly elevated sympathetic nerve density, driven by tumor cell‐derived NGF. Itami et al. discovered that NGF expression levels correlate with increased sympathetic nerve density in the tumor mass and a poorer prognosis in patients. NGF inhibition attenuated nerve sprouting and tumor malignancy in gastric cancer [101]. Qi et al. discovered that sympathetic nerves participate in promoting gastric cancer metastasis by activating β2‐AR/STAT3/ERK signaling [102]. In colorectal cancer (CRC), sympathetic nerves promote tumor progression via NE‐mediated activation of Adra2a/LATS1/YAP signaling [103]. In small cell lung cancer, sympathetic nerve‐derived NE activates β2‐adrenergic receptor (ADRB2)/protein kinase A (PKA)/pCREB signaling to drive tumor growth [104]. Infection with the Epstein–Barr virus (EBV) is a risk factor for various types of tumors [105]. A recent study demonstrated that sympathetic nerves contribute to EBV‐mediated tumor progression [106]. EBV infection promotes sympathetic nerve infiltration into diffuse large B‐cell lymphoma (DLBCL). Reciprocally, these nerves enhance the proliferation of EBV‐positive DLBCL cells through adrenergic signaling [106]. Adrenergic signaling regulates malignant progression across multiple cancer types through diverse mechanisms. Targeting this neuro‐regulatory axis is a promising therapeutic strategy for disrupting tumor–nerve crosstalk in oncology.

Ach, another well‐known neurotransmitter with a critical role at synapses and the neuromuscular junctions, plays a critical role in mediating parasympathetic nerve signaling [107]. Nicotinic acetylcholine receptors (nAChRs) and metabotropic G protein‐coupled receptors called muscarinic acetylcholine receptors (mAChRs) are essential effectors of Ach signaling [108]. In addition to neuronal cells, nAChRs and mAChRs are expressed in diverse non‐neuronal cell types, including lymphocytes, macrophages, endothelial cells, and epithelial cells, indicating their broader functions beyond synaptic transmission. Emerging evidence supports the role of ACh's in regulating fundamental cellular processes, including proliferation, differentiation, apoptosis, and immune responses [109]. ACh has been identified as a critical regulator of primary tumor growth and metastatic progression [110]. Tumor cells express both nAChRs and mAChRs, enabling them to respond to parasympathetic nerve‐derived signals. In prostate cancer, Ach signaling promotes tumorigenesis and progression by activating the CHRM1 receptor. CHRM1 agonism improves tumor cell invasion into the pelvic lymph nodes (LN), while CHRM1 antagonists effectively suppress LN metastasis [6]. Zhao et al. discovered that greater vagal innervation of the gastric lesser curvature is associated with a higher incidence of tumors, and surgical denervation of the vagus nerve attenuates tumorigenesis and enhances the effect of chemotherapy in gastric cancer, establishing the vagus nerve as a key regulator of gastric cancer [7]. Mechanistically, vagus nerve‐derived Ach activates Wnt signaling in gastric cancer cells via muscarinic acetylcholine receptor M3 (CHRM3) [7]. Similar results was obtained by Wang et al. that CHRM3 is involved in mediating vagus nerve‐induced gastric cancer progression [111]. Hayakawa et al. discovered that NGF production in gastric epithelial cells is stimulated by CHRM3‐dependent cholinergic signaling, which is activated by Chat^+^ tuft cell‐derived Ach during the early phase of tumorigenesis or by vagus nerve‐derived Ach during the later phase of tumorigenesis. NGF further promotes axonogenesis, increasing the density of vagus nerve in gastric cancer, forming a positive feedback loop between Ach‐NGF to promote gastric cancer initiation by activating YAP signaling [67]. A recent clinical study demonstrated that therapeutic vagotomy improves survival outcomes in patients with locally advanced unresectable gastric cancer by eliminating parasympathetic innervation, highlighting the potential application of vagotomy as a therapeutic strategy in gastric cancer treatment [112]. Our investigations research in cancer neuroscience revealed that intrahepatic cholangiocarcinoma (ICC) exhibits significant parasympathetic hyperinnervation. Using comprehensive molecular profiling and functional studies, we identified that parasympathetic nerve‐derived Ach promotes ICC progression and induces chemotherapy resistance via the α5 nicotinic acetylcholine receptor (CHRNA5)‐mediated activation of CAMKII/GSK3β/β‐catenin signaling [12]. Several studies have identified cholinergic signaling as a tumor‐suppressive pathway in PDAC. Partecke et al. reported that subdiaphragmatic vagotomy promotes tumor growth and invasive features of tumor cells, revealing a protective role of parasympathetic innervation against cancer progression [113]. Furthermore, Renz et al. demonstrated that activation of cholinergic signaling activation suppresses cancer stemness and tumor growth in genetically engineered PDAC models through CHRM1‐mediated inhibition of both the MAPK/EGFR and PI3K/AKT pathways [114]. However, Zhang et al. made a counterintuitive observation that high‐grade VAChT‐positive parasympathetic neurogenesis (>15 fibers/[5 × 0.785] mm) shared a close association with the poor prognosis of patients with PDAC [115]. Early studies demonstrated that nicotine, a nAChR agonist, promotes tumor initiation and progression in PDAC [116, 117, 118], indicating that parasympathetic nerve‐derived Ach may similarly accelerate tumor growth through nAChR activation. These conflicting findings regarding cholinergic signaling in PDAC likely reflect methodological differences across preclinical models. The translational validity of Partecke et al.’s findings remains questionable due to methodological limitations. While the murine model demonstrated that subdiaphragmatic vagotomy accelerated pancreatic tumor progression, the study lacked quantitative tumor burden analysis (relying solely on representative images) to demonstrate statistical significance, and they did not identify specific cholinergic receptors response for these effects. Conversely, Renz et al. provided robust evidence through quantitative measurements of the effect of vagotomy on tumor burden and identified CHRM1 as the key receptor suppressing tumor growth. However, nAChR involvement was not investigated in this study. This reveals a potential duality in cholinergic regulation: While the CHRM1 receptor appears to be tumor‐suppressive, nAChRs may promote tumor progression. Clinical observation of poor prognosis with high vagal innervation in patients with PDAC supports this dichotomy. Concerns have been raised regarding the translational validity of results obtained from current preclinical models, highlighting the need for more clinically relevant experimental systems to determine the precise role of parasympathetic innervation in PDAC progression. Emerging evidence has established the functional significance of ACh and AChRs in diverse malignancies, including breast, lung, liver, and head and neck cancers [119, 120, 121]. While these findings demonstrate that AChR‐expressing tumor cells may respond to parasympathetic regulation, direct experimental evidence for parasympathetic neural control of cancer progression is limited. Further research is required to provide convincing evidence regarding the regulatory role of parasympathetic nerves in tumors.

Sensory nerves represent a critical component of cancer neuroscience, particularly in mediating cancer‐associated pain. Sensory neuron‐derived calcitonin gene‐related peptide (CGRP) promotes pain pathogenesis by stimulating vasodilation‐mediated accumulation of proinflammatory factors, which irritate peripheral nociceptors, and induce hyperalgesia and allodynia [122]. As one of the most common sites of cancer metastasis, bone is highly infiltrated by sensory nerves. Park et al. reported that prostate cancer cells metastasizing to bone induce the sprouting of sensory nerves, creating a feedforward loop in which nerve‐derived CGRP activates the CRLR/p38/HSP27 signaling axis, promoting tumor cell proliferation [123]. This reciprocal interaction between cancer cells and sensory nerves promotes metastatic colonization of bone and the development of cancer‐induced bone pain. Moreover, sensory neurons play a pivotal role in the pathogenesis of pancreatitis [124], and generalized inflammation is required for the development of precancerous pancreatic intraepithelial neoplasia [125]. Saloman et al. discovered that ablation of sensory neurons suppresses pancreatic tumor initiation and progression by reducing neurogenic inflammation in the TME [8]. Furthermore, sensory nerves promote TNBC invasion and metastasis by activating the semaphorin 5A/PlexinB3 signaling axis [126]. Peterson et al. reported that cutaneous nerves regulate basal cell carcinoma (BCC) progression, and sensory denervation inactivates Hedgehog signaling, attenuating BCC tumor growth [127]. Zhang et al. discovered that nociceptive nerve‐derived CGRP enables cancer cells to survive under glucose‐deprived conditions by inducing cytoprotective autophagy. This process is mediated through Rap1‐dependent disruption of mTOR‐Raptor interactions, revealing a novel neuro‐mediated metabolic adaptation mechanism in tumors [128].

Serotonergic neurons, a crucial component of the peripheral nervous system, play significant roles in regulating physical activity by secreting serotonin, also known as 5‐hydroxytryptamine (5‐HT). Moreover, cancer cells express 5‐HT receptors to respond to serotonergic neuronal signaling. Zhu et al. reported that CRC‐associated microbiota metabolite isovalerate activates enteric serotonergic neurons to produce 5‐HT, which subsequently enhances cancer stemness via 5‐HT receptor‐mediated Wnt/β‐catenin activation, thereby driving tumor progression. Blocking 5‐HT signaling significantly suppresses CRC tumorigenesis and metastasis [129]. In addition to directly activating 5‐HT receptors, 5‐HT can regulate tumor progression by inducing histone serotonylation. A recent study revealed that serotonergic neurons‐derived 5‐HT suppresses ependymoma tumor progression by inducing serotonylation of histone H3, which promotes ETV5 expression and inhibits NPY expression, revealing a novel neurotransmitter‐mediated epigenetic mechanism in cancer treatment [130].

#### 5.1.2 Metabolic Plasticity‐Mediated Regulatory Effect of Nerves on Tumor Progression

Metabolic plasticity, the adaptive ability of cancer cells to alter their metabolic programming, is now recognized as essential for successful metastatic progression, enabling tumor cells to navigate each step of the metastatic cascade [131]. Tumor cells require sufficient nutrients to meet their proliferative demands. They use metabolic plasticity to adapt and survive in harsh microenvironments by upregulating scavenging pathways, such as autophagy and micropinocytosis, and by utilizing metabolites released from stromal fibroblasts and macrophages [132]. Neurons release amino acid‐derived neurotransmitters, including Gly and D‐serine (Ser), indicating potential metabolic support for PDAC cells in nutrient‐deprived microenvironments through neurotransmitter recycling [133]. Banh et al. discovered that nerves could secrete Ser to sustain the growth of extracellular Ser‐dependent PDAC cells in Ser/Gly‐deprived conditions [134]. They observed that PDAC cells upregulate NGF expression to promote tumor innervation in Ser/Gly deprivation. Ser‐deprivation causes ribosomal stalling at two of the six Ser codons, TCC and TCT, allowing the selective translation and secretion of NGF [134]. Peripheral axons release Ser to maintain PDAC cell proliferation during Ser/Gly deprivation. This reveals a novel neurometabolic axis in which nerve‐derived metabolites support tumor survival under nutrient stress [134].

To achieve metabolic plasticity, cancer cells undergo autonomous adaption to modify their metabolic program and perform metabolic rewiring by regulating the expression of metabolic enzymes and metabolite concentrations. A recent study revealed that the metabolic plasticity of cancer cells can also be achieved through interaction with nerves [135]. This study revealed that, when cocultured with cancer cells, neurons undergo metabolic reprogramming characterized by increased mitochondrial biogenesis. These neuron‐derived mitochondria were transferred to adjacent cancer cells, resulting in enhanced mitochondrial function, including elevated basal, maximal, and spare respiratory capacity in the recipient breast cancer cells [135]. Using precise lineage tracing, researchers have demonstrated that cancer cells acquiring neuron‐derived mitochondria exhibit significantly enhanced metabolic plasticity and stress resistance. These transferred mitochondria promote metastatic competence by enabling recipient cancer cells to survive in harsh microenvironments and proliferate at distant sites [135]. While these studies indicate neural regulation of tumor metabolic plasticity during cancer progression, the full scope of nerve‐derived metabolic contributions remains incompletely characterized. Further research is required to quantify neural metabolic inputs across tumor types and identify additional nerve‐secreted metabolites that may influence oncogenic metabolism.

### 5.2 Indirect Regulatory Effect of the Nerve System on Tumor Progression by Modulating TME

**FIGURE 4:**
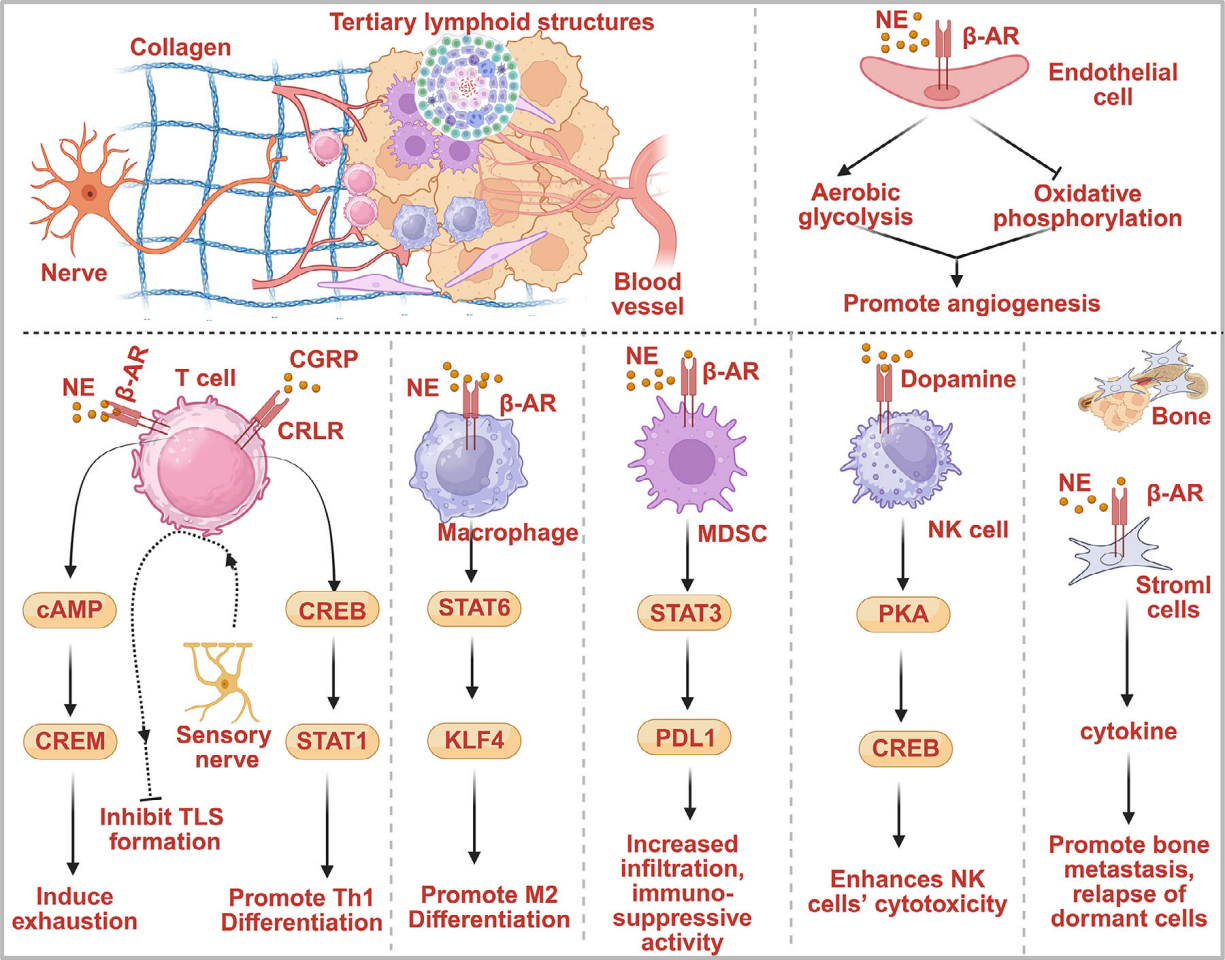
Nerves participate in regulating the TME. Nerves could secrete neurotransmitters to target corresponding receptors in stromal cells, including T cells, macrophages, MDSCs, NK cells, and endothelial cells, to regulate the malignant phenotype of tumors. (This figure was created using BioRender.com.)

The TME, composed of ECM and various stromal cell types, such as fibroblasts, endothelial cells, and immune cells, plays a crucial role in regulating tumor progression [136]. Emerging evidence demonstrates that nerves participate in regulating angiogenesis, immune cell infiltration and polarization, and remodeling the TME to regulate tumor progression. The mechanisms underlying neural regulation of TME dynamics are discussed in this section (Figure 4).

Immune cells are crucial components of the TME. Tumor‐educated immune cells progressively lose their immunosurveillance capacity and often adopt immunosuppressive phenotypes, thereby establishing an immune‐permissive niche for tumor escape. Neural signaling may indirectly shape the tumor immune microenvironment by modulating the cancer cell‐mediated immune responses. For example, interleukin‐6 (IL‐6) is an important proinflammatory cytokine involved in lymphocyte activation. Norepinephrine‐mediated activation of ADRB2 signaling could stimulate IL‐6 expression in ovarian tumors, suggesting the potential of sympathetic nerves in regulating the tumor immune microenvironment through the ADRB2/IL‐6 signaling axis [137, 138, 139]. Furthermore, Yang et al. reported that PDAC tissues with PNI exhibit an increased concentration of Ach, which impairs the ability of PDAC cells to recruit CD8^+^ T cells via HDAC1‐mediated suppression of CCL5, fostering an immune‐permissive microenvironment for tumor progression [140]. In addition to the indirect regulatory effect of nerves on immune cells infiltration by modulating the generation of chemokines and cytokines in cancer cells, the direct action of nerves on immune cells can be achieved by activating neural receptors in immune cells. Pioneering work by Hugo et al. in the 1980s proposed neural regulation of immune homeostasis through negative feedback mechanisms, foreshadowing our current understanding of neuro–immune crosstalk in cancer [141]. In case of pathogen challenge, immune cells release cytokines that cross the BBB, activating the hypothalamic–pituitary–adrenal axis to generate systemic immune responses [142]. Beyond systemic neurohormonal effects, recent research has demonstrated that the CNS mediates systemic anti‐inflammatory responses through vagus nerve‐mediated parasympathetic efferent signaling [143]. For example, splenic adrenergic nerves activate ADRB2‐expressing T cells to release Ach, which subsequently suppresses TNF production in nAChR‐expressing macrophages through paracrine signaling [144]. The inflammatory reflex, a neuro–immune circuit, established neural modulation as a viable anti‐inflammatory therapeutic strategy [145]. Emerging research demonstrates that autonomic nerves regulate immune cell recruitment and differentiation within the TME [146].

#### 5.2.1 Neural Regulation of T Cell Activity

CD8^+^ cytotoxic T cells, a subset of immune cells capable of recognizing and killing cancer cells, are the most potent effectors of the anticancer immune response and the backbone of cancer immunotherapy [147, 148]. CD8^+^ T cells express functional adrenergic receptors, enabling their antitumor activity to be modulated by sympathetic nervous system signaling. ADRB1 has been identified as a neural‐regulated immune checkpoint that promotes T cell exhaustion in esophageal squamous cell carcinoma, revealing a novel mechanism of neuroimmune‐mediated immunosuppression in the TME [149]. Chen et al. reported that denervation of the sciatic nerve, which innervates the popliteal LN, induces the expression of IFN‐γ in LN CD8^+^ T cells as a result of inactivation of ADRB2, thereby establishing sympathetic nerves as direct regulators of CD8^+^ T cell effector function [150]. This neural regulatory mechanism extends to tumor‐infiltrating CD8^+^ T cells, where sympathetic signaling modulates their antitumor activity in the TME. Globig et al. [151] observed that the exhausted CD8^+^ T cells preferentially localize near sympathetic nerve fibers, a phenomenon mediated by activation of ADRB1 in T cells. Sympathetic nerves‐derived catecholamine directly targets ADRB1‐expressing CD8^+^ T cells, impairing T cell proliferation, cytokine production, and inducing T cell exhaustion via ADRB1–cAMP–CREM signaling axis. β‐Blocker and immune checkpoint blockade (ICB) combination therapy synergistically enhanced CD8^+^ T cell responses and antitumor efficacy in PDAC, indicating that adrenergic receptor modulation is a promising strategy to improve ICB efficacy.

In addition to sympathetic nerves, sensory nerves participate in regulating T cell activity in tumors. Balood et al. discovered that nociceptor neuron‐derived CGRP induces CD8^+^ T cell exhaustion via receptor activity‐modifying protein 1 (RAMP1) activation, thereby accelerating tumor progression. Therapeutic targeting of the CGRP/RAMP1 axis reduced T cell exhaustion and melanoma growth, highlighting sensory nerves as key regulators of antitumor immunity [152]. Furthermore, sensory nerve‐secreted CGRP can reduce the infiltration and activity of T helper type 1 (Th1) CD4^+^ and CD8^+^ T cells, thereby modulating the antitumor immune activity and promoting tumor growth [153]. The balance between Th1 cells and other Th cells is critical for antitumor response [154]. The equilibrium between Th1 and other Th subsets is essential for effective antitumor immunity. Emerging evidence indicates that CGRP‐secreting sensory nerves may regulate this balance by modulating Th1 cell differentiation in the TME. Hou et al. discovered that CGRP‐secreting nerves are involved in Th cell differentiation via the CGRP–RAMP3–cAMP–STAT1 signaling axis [155]. Tertiary lymphoid structures (TLS) have emerged as reliable biomarkers for predicting favorable clinical outcomes in cancer patients, particularly in those undergoing immunotherapy [156, 157, 158]. Vats et al. discovered that sensory nerves modulate effector function of tumor‐infiltrating lymphocytes, thereby regulating melanoma growth [159]. Their work demonstrated that sensory nerve ablation promotes the maturation of intratumor high endothelial venules and the formation of TLS with increased dense clusters of T and B cells. Moreover, sensory nerve ablation augments the influx of myeloid and lymphoid cells into the tumor, expands the intratumoral BCR IgH repertoire, and primes T cells. These findings establish tumor‐associated sensory nerves as suppressors of protective antitumor immune responses in the TME [159].

These findings establish neural regulation of T cell function as a key mechanism of tumor immune evasion. Therapeutic targeting of neuroimmune signaling pathways is a promising strategy for improving antitumor immunity by restoring T cell effector function in the TME.

#### 5.2.2 Neural Regulation of Tumor‐Associated Macrophages’ Activity

Tumor‐associated macrophages (TAMs) are pivotal stromal components that orchestrate multiple oncogenic processes in the TME [160]. Neural signaling modulates macrophage recruitment and polarization in the TME. CD163^+^ TAMs promote tumor progression by inhibiting T cell‐mediated antitumor immunity through numerous immunosuppressive mechanisms [161]. Guillot et al. discovered that denervation of sympathetic nerves increased CD163^+^ TAM infiltration in PDAC, suggesting a protective role of sympathetic nerves in limiting protumorigenic TAM accumulation [162]. Another study reported that adrenergic signaling activation increases macrophage infiltration into primary tumor parenchyma and drives the M2 polarization of these macrophages via activating the ADRB2, thereby facilitating the distant metastasis of breast cancer. Correspondingly, β‐adrenergic blockade with propranolol inhibits this prometastatic pathway [163]. A recent study reported that ADRB2 activation induces Kruppel‐like transcription factor 4 expression through JAK1/STAT6 signaling, thereby driving M2 macrophage polarization and promoting non‐small cell lung cancer [164].

While neural regulation of macrophage activity has been well‐characterized in non‐neoplastic conditions [165], evidence for direct neuro–macrophage crosstalk in tumors remains limited. Further research is required to determine whether similar regulatory mechanisms operate within the TME.

#### 5.2.3 Neural Regulation of Myeloid‐Derived Suppressor Cells’ Activity

Myeloid‐derived suppressor cells (MDSCs), a diverse population of cells derived from immature myeloid cells, play a significant role in immunosuppression and are associated with resistance to anticancer therapies, especially immunotherapies, in various types of tumors [166]. MDSCs generate an immune suppressive microenvironment by producing inducible nitric oxide synthase, arginase 1, transforming growth factor‐beta, IL‐10, cyclooxygenase‐2, and indoleamine 2,3‐dioxygenase, as well as generating Fas‐ligand to induce apoptosis of Fas‐expressing TILs [166, 167]. Mohammadpour et al. discovered that MDSCs express ADRB2, and activation of ADRB2 regulates MDSC frequency and survival in breast cancer tissues. Moreover, ADRB2 signaling regulates the immunosuppressive activity of MDSCs via STAT3‐mediated modulation of immunosuppressive molecules, such as arginase‐1 and PD‐L1, suggesting a potential role for sympathetic nerves in modulating the immune microenvironment in breast cancer [168]. A recent study reported that various types of tumors, including lung, prostate, colon, and breast cancer cells, secrete leukemia inhibitory factor and galectin‐3, which activate the paraventricular nucleus of the hypothalamus, a brain region initiating efferent sympathetic signals. This stimulation triggers sympathetic outflow, activating peripheral ADRB2, promoting MDSC infiltration and their immunosuppressive activity [169].

#### 5.2.4 Neural Regulation of Natural Killer Cells Activity

Natural killer (NK) cells are critical innate immune regulators that play pivotal roles in physiological homeostasis and pathological processes [170]. Emerging evidence indicates neural regulation of the antitumor activity of NK cells. An early study revealed that the enriched environment, characterized by a rodent model of “eustress” or positive stress [171], promotes NK cell infiltration into tumors and increases NK cells anticancer activity through mechanism involving sympathetic nervous system‐mediated β‐adrenergic signaling activation and CCR5 overexpression in NK cells, thereby inhibiting tumor progression [172]. Previous studies revealed that electrical stimulation of the sciatic nerve modulates immune function by releasing catecholamine [173, 174]. Li et al. discovered that sciatic nerve stimulation increases NK cell cytotoxicity through dopamine receptor‐mediated activation of the cAMP–PKA–CREB signaling pathway, effectively suppressing breast tumor growth. Furthermore, neurostimulation upregulates tumor PD‐L1 expression via IFN‐γ‐dependent mechanisms. Combining sciatic nerve stimulation with anti‐PD‐1 therapy synergistically improved tumor control compared with monotherapies [175]. These findings demonstrate that dopamine improves the efficiency of anti‐PD1 therapy by potentiating NK cell activity. In addition to direct neural regulation, NK cell function can also be indirectly modulated through neuro–stromal crosstalk in the TME. CAFs‐derived NGF can activate nociceptor neurons to secrete CGRP, which activates RAMP1 to inhibit IL‐15 expression in CAFs, thereby suppressing NK cell infiltration and cytotoxicity. This neuro–stromal signaling axis promotes PDAC progression [176].

While the role of NK cells in cancer neuroscience remains understudied, emerging evidence demonstrates their dual therapeutic potential. Previous studies have identified NK cells as mediators of neuropathic pain resolution [177]. This raises the intriguing possibility that neural‐activated NK cells may simultaneously suppress tumor progression and alleviate cancer‐associated pain, which warrants further investigation.

#### 5.2.5 Neural Regulation of Tumor Bone Metastasis

Bone metastasis is a common and fatal complication of osteophilic tumors, including breast, prostate, and lung cancers, and severely affects the patient's quality of life and life expectancy [178]. Moreover, bone is highly innervated by sensory and sympathetic nerves [179]. Emerging evidence demonstrates that skeletal sympathetic innervation regulates bone metastasis. Systematic adrenergic signaling, activated by psychosocial factors, establishes a permissive microenvironment that facilitates metastatic colonization of osteotropic tumors [180]. Sympathetic nerves infiltrating bone release NE, which directly activates adrenergic receptor signaling in the bone marrow stroma cells. This neuro–stromal interaction establishes a prometastatic niche that promotes anoikis resistance, metastatic colonization, and subsequent tumor growth, thereby facilitating bone metastasis in osteotropic malignancies. Campbell et al. discovered that bone‐infiltrating sympathetic nerves release norepinephrine, activating ADRB2‐expressing bone marrow stroma osteoblasts, subsequently upregulating RANKL expression and promoting cancer cell migration toward bone [181]. Complementing these findings, Decker et al. reported that sympathetic nerves in the bone marrow secrete norepinephrine, which reactivates dormant metastatic prostate cancer cells and adjacent niche cells through adrenergic signaling, driving disease recurrence in bone metastases [182].

#### 5.2.6 Neural Regulation of Tumor Angiogenesis

Blood vessels are crucial regulators of tissue homeostasis by facilitating nutrient, metabolite, and oxygen exchange. To support their rapid proliferation, cancer cells secrete proangiogenic factors that drive pathological angiogenesis, establishing an extensive vascular network within tumors [183]. Unlike the well‐organized vasculature of normal tissues, tumor‐associated neovascularization exhibits defective architecture characterized by incomplete pericytes, endothelial cells, and basement membrane coverage. These structurally abnormal vessels exhibit tortuous, dilated, and irregular morphology that facilitates metastatic dissemination [184, 185]. Antiangiogenic therapy, which targets tumor neovascularization, is the cornerstone of current anticancer treatment strategies. Sympathetic nerves, which anatomically colocalize with blood vessels, regulate vascular tone through norepinephrine‐mediated contraction of vascular smooth muscle cells. During development, sympathetic nerves grow in parallel with blood vessels and significantly modulate vascular patterning and maturation [186, 187]. Recent research demonstrates that nerves regulate tumor angiogenesis, mirroring their role in physiological vascularization. Recent research revealed that adrenergic nerves infiltrating the prostate cancer microenvironment release norepinephrine, activating ADRB2‐expressing endothelial cells. This neural signaling induces endothelial cells to undergo metabolic reprogramming characterized by suppressed oxidative phosphorylation and enhanced aerobic glycolysis, ultimately driving protumorigenic angiogenic bursts that accelerate cancer progression [56]. Besides, adrenergic nerves may indirectly promote tumor angiogenesis by activating the cAMP–PKA signaling pathway in cancer cells, thereby increasing VEGF production and secretion [89]. These findings highlight the pivotal role of neural regulation in tumor vascularization.

## 6 Therapeutic Applications

Recent advances have significantly expanded our understanding of neuro–tumor interactions, indicating sophisticated crosstalk between peripheral nerves and cancer and stromal cells. These neural inputs regulate tumor growth, metastasis, angiogenesis, immune evasion, and metabolic reprogramming through neurotransmitter and neuropeptide signaling. The rapidly evolving mechanistic insights into tumor–nerve interactions highlight promising therapeutic opportunities for molecularly targeted interventions that disrupt protumorigenic neural signaling.

Nerves contribute to tumor progression through numerous mechanisms (Table 1). Signaling molecules, including neurochemicals or NGFs, such as norepinephrine, Ach, NGF, and BDNF, are attractive therapeutic targets. Drug repurposing, the application of existing medications for cancer treatment, has emerged as a valuable strategy for developing novel antitumor therapies [188]. Considering the regulatory role of neurochemicals and NGFs in the malignant phenotype of tumors, and the availability of targeted pharmacological modulators, these neural signaling molecules represent promising therapeutic targets. Preclinical studies have demonstrated that adrenergic signaling drives the progression of various cancer types, and some clinical evidence indicates a close association between β‐blocker use and improved oncological outcomes [189, 190], indicating the significant potential of adrenergic receptor antagonists in the treatment of cancer patients. The therapeutic potential of adrenergic antagonists in oncology has been extensively reviewed [87].

**TABLE 1:** The regulatory role of nerves in the malignant phenotype of tumors and the underlying mechanisms.

Cancer Type	Nerves	Phenotype	Mechanism	References
Breast cancer	Sympathetic nerves	Promote bone metastasis,	β2AR/RANKL axis	[181]
Breast cancer	Sympathetic nerves	Influence MDSC frequency and survival in breast cancer tissues and immunosuppressive activity	β2‐AR/STAT3 signaling	[168]
Breast cancer	Sympathetic nerves	Promote distant metastasis	Increase the infiltration of macrophages into primary tumor parenchyma and stimulate the M2 polarization of these macrophages via activating the β2‐adrenergic receptor in macrophage	[163]
Breast cancer	Sensory nerves	Stimulate cancer invasion and metastasis	Secrete semaphorin 5a (Sema5A) to interact with PlexinB3 in cancer cell	[126]
Pancreatic ductal adenocarcinoma	Sympathetic nerves	Inhibit tumor growth and spread.	Reducing the infiltration of CD163+ TAM	[162]
Pancreatic ductal adenocarcinoma	Sympathetic nerves	Reduce ICB sensitivity	Induce CD8 cell exhaustion	[151]
Pancreatic ductal adenocarcinoma	Parasympathetic nerve	Promote tumor growth	Impair the ability of PDAC cells to recruit CD8+ T cells via HDAC1‐mediated suppression of CCL5 and directly inhibited IFN‐γ production of CD8+ T cells	[140]
Pancreatic ductal adenocarcinoma	Parasympathetic nerve	Inhibit stemness property and tumor growth	CHRM1‐mediated inhibition of MAPK/EGFR and PI3K/Akt signaling pathway	[114]
Pancreatic ductal adenocarcinoma	Sensory neurons	Stimulate initiation and progression of cancer		[8]
Prostate cancer	Sympathetic nerves	Promote a burst of tumor angiogenesis that fuels cancer progression	Stimulate the β2AR‐expressing endothelial cells to undergo metabolic reprogramming toward the inhibition of oxidative phosphorylation	[56]
Prostate cancer	Parasympathetic nerve	Promote metastasis	Activating CHRM1 receptor in prostate cancer cells	[6]
Gastric cancer	Sympathetic nerves	Promote metastasis	Activating β2‐AR/STAT3/ERK signaling	[102]
Gastric cancer	Vagus nerve	Promote tumor initiation and progression and stemness property	M3 receptor/Wnt signaling, M3 receptor/YAP signaling	[7] (67)
Ovarian tumor	TRPV1 positive nerve	Potentiate tumor growth and contribute to treatment resistance		[58]
Ovarian tumor	β‐Adrenergic signaling	Stimulate angiogenesis	cAMP–PKA/VEGF signaling	[89]
Cholangiocarcinoma carcinoma	Parasympathetic nerve	Promote tumor metastasis and chemotherapy‐resistance	Acetylcholine/CHRNA5 axis‐mediated activation CAMKII/GSK3β/β‐catenin signaling	[12]
Basal cell carcinoma	Sensory nerve	Promote tumor initiation and progression and stemness property	Activating Hedgehog signaling	[127]
Head and neck squamous cell carcinoma	Sensory nerves	Accelerating tumor growth	Secreting CGRP to acting on the adaptive immune system to decrease Th1 CD4 T cells and activated CD8 T cells in the TME	[153]
Colorectal carcinoma	Sympathetic nerve	Stimulate tumor progression	NE‐mediated activation of Adra2a/LATS1/YAP	[103]
Melanoma	Sensory nerves	Impede the formation of tertiary lymphoid structures		[159]
Oral cancer	Sensory nerves	Help cancer cells to thrive in nutrient‐poor environments	CGRP induces cytoprotective autophagy in cancer cells via Rap1‐mediated disruption of the mTOR/Raptor interaction	[128]

### 6.1 Integrating Neuroactive Drugs With Conventional Therapies

Opportunities exist for repurposing these neuroactive drugs in combination with immune checkpoint inhibitors or cytotoxic therapies to improve antitumor efficacy. Some studies have evaluated the efficiency of combining nerve signaling interventions with conventional therapies. Moreover, several studies revealed that chemotherapy induces infiltration of sympathetic nerves into tumors, which amplifies adrenergic signaling to facilitate cancer survival and promote cancer progression [100, 191]. Blocking adrenergic signaling might be effective strategy for improving the effects of chemotherapy. Zuo et al. developed NGF‐targeted small interfering RNA (siRNA) delivered via poly (lactic‐co‐glycolic acid) (PLGA) nanoparticles for breast cancer therapy. This approach effectively suppressed chemotherapy‐induced sympathetic nerve proliferation by inhibiting NGF signaling, resulting in improved antitumor efficacy [100]. Zhou et al. observed the dose‐limiting toxicity of propranolol in murine models and subsequently developed a pH‐sensitive liposomal formulation that selectively released propranolol in the acidic TME. This targeted delivery system demonstrated improved safety profiles while maintaining therapeutic efficacy [192]. Propranolol‐containing liposomes effectively inhibit adrenergic signaling and exert synergistic antitumor effects with chemotherapy drugs in several cancers, including prostatic cancer, PDAC, and melanoma [192]. In addition to chemotherapy, neural signaling modulation synergizes with immune checkpoint inhibitors. For example, ADRB antagonists remodel the tumor immune microenvironment by improving T cell recruitment and activation, thereby potentiating immunotherapy efficacy [151, 193, 194]. Fjæstad et al. discovered that propranolol enhanced the therapeutic efficacy of anti‐CTLA‐4 immunotherapy in patients with soft tissue sarcoma, highlighting the potential of β‐adrenergic receptor blockade as an immune checkpoint inhibitor adjuvant [193]. Tumor‐innervating peripheral nerves contribute to an immunosuppressive environment. Targeting these neural–immune interactions is a promising strategy for improving ICB efficacy and clinical outcomes. Advances in single‐cell sequencing technologies could enable the characterization of the role of neural signaling in immune cell reprogramming, facilitating the development of novel antitumor immunotherapy strategies.

### 6.2 Anticancer Therapy Based on Pain‐Modulating Molecules

Patients with cancer commonly experience cancer‐associated pain at the primary and metastatic sites, which caused by increased perineural infiltration and tumor‐induced hyperalgesia/allodynia. The cancer pain‐modulating molecules also promote tumor growth, making them dual‐purpose therapeutic targets for pain management and antitumor therapy. NGF is a key regulator of pain signaling and local tissue nociception [195, 196]. NGF‐targeting antibodies and neutralizing medicines have been extensively evaluated for their ability to modulate pain in preclinical studies. Clinical studies have demonstrated that NGF inhibitors outperform nonsteroidal anti‐inflammatory drugs NSAIDs in the management of chronic pain conditions, including osteoarthritis and chronic low back pain [197]. In a phase 3 clinical trial, the NGF‐targeting monoclonal antibody, tanezumab, demonstrated significant analgesic efficacy for bone metastasis‐related pain, indicating its potential as a novel therapeutic option for cancer pain management [198]. Moreover, the transient receptor potential vanilloid‐1 (TRPV1) receptor is a promising dual‐purpose target for cancer therapy, as it promotes tumor progression while also mediating cancer‐associated pain. Preclinical studies have demonstrated that resiniferatoxin (RTX)‐induced sensory neuron denervation simultaneously suppresses pancreatic cancer progression and alleviates neuropathic pain [199, 200]. Clinical studies evaluating RTX for the treatment of patients with severe or refractory cancer‐associated pain are currently underway [201].

### 6.3 Locally Targeted Delivery of Neural Signaling‐Targeting Agents

The systemic application of neural signaling‐targeting agents for treating tumors might cause severe unintended complications. For example, RTX, a potent TRPV1 agonist used for nociceptor ablation, may induce off‐target complications due to widespread TRPV1 expression, including hypothermia, cardiovascular stimulation, respiratory disturbances, and nonselective neurotoxicity [200, 202, 203]. Additionally, the systematic application of propranolol, an approved drug with relatively good safety profiles, poses significant clinical risks due to BBB‐associated neurocognitive effects, cardiorespiratory and metabolic disturbances and neurobehavioral alterations including sleep architecture disruption and memory impairment [204, 205, 206]. Locally targeted delivery systems, particularly tumor‐specific nanoparticles carrying neural‐modulating agents, prolong drug release to increase tumor‐specific accumulation and minimize systemic exposure, thereby preventing adverse effects; this represents a promising strategy for the clinical translation of neuro‐targeted cancer therapies [192, 207]. Yang et al. developed a tumor membrane‐coated nanoplatform for codelivery of CpG adjuvant and propranolol, which enhances priming of naïve CD8^+^ T cells and promotes effector T cell egress from LNs. Furthermore, it can alter the immunosuppressive TME by reducing regulatory cell populations while increasing infiltration of B and NK cells, thereby inhibiting melanoma tumor growth [208]. As previously stated, breast cancer‐targeted PLGA nanoparticles containing siRNA targeting NGF suppress NGF‐mediated proliferation of sympathetic nerves post‐chemotherapy, thereby demonstrating enhanced anticancer efficiency [100]. We developed a nerve‐targeting nanocarrier, Lar@NP‐OMVs by conjugating the nerve‐binding peptide NP41 and the Trk inhibitor larotrectinib with Escherichia coli Nissle 1917‐derived outer membrane vesicles. Lar@NP‐OMVs treatment significantly inhibited neurite outgrowth by disrupting the neurotrophin/Trk signaling pathway and inducing M2‐like TAM‐mediated nerve injury, thereby suppressing neural‐induced tumor proliferation, migration, and angiogenesis, and enhancing chemotherapy efficacy in pancreatic cancer [209]. Li et al. discovered that nerve‐derived glutamate activates the N‐methyl‐d‐aspartate receptor (NMDAR), which promotes pancreatic cancer progression and PNI through CaMKII/ERK–MAPK signaling. Based on this discovery, they developed the IONPs–PEG–scFvCD44v6–scAbNMDAR2B nanoparticles that target both CD44 variant isoform 6 and the NMDAR subunit 2B on PDAC cells, thereby inhibiting pancreatic tumor growth and PNI in vivo [210]. Lipid‐based drug delivery systems have emerged as a transformative therapeutic platform for osseous complications, offering precise bone targeting with minimal off‐tissue effects. These nanosystems facilitate the targeted delivery of diverse therapeutic payloads, including small molecules, nucleic acids, and peptides, demonstrating dual efficacy in inhibiting bone metastasis progression and alleviating cancer‐induced pain through neural circuit modulation within the bone microenvironment [211].

## 7 Conclusion and Future Directions

After decades of genetic investigations on tumorigenesis, the crucial role of the TME has become increasingly clear. Mounting evidence has revealed the interaction between cancer and nerves, opening new area of cancer neurobiology. On one hand, cancer cells can hijack and remodel nervous system structure and function; on the other hand, neural signaling reciprocally regulates tumor growth both directly and indirectly, through microenvironmental modulation. Neural signaling mediated by neurotransmitters and neuropeptides plays a crucial role in tumor progression. Targeting these neural circuits is a novel therapeutic paradigm, and elucidating their mechanisms could accelerate clinical translation of neural‐modulating anticancer strategies. Repurposing of existing neuroactive drugs, including selective β‐blockers, antianxiety, and antiepileptic drugs, represents a promising translational approach in cancer neuroscience. A series of clinical trials evaluating the efficacy of neural signaling targeting‐based anticancer strategies has been conducted (Tables 2 and 3).

**TABLE 2:** Completed clinical trials targeting the neuro–cancer crosstalk.

Study name	Reference	Drug	Targets	Outcome
Perioperative COX‐2 and beta‐adrenergic blockade improves metastatic biomarkers in breast cancer patients in a phase‐II randomized trial	[212]	Propanolol etodolac	NE COX‐2	Perioperative inhibition of COX‐2 and β‐adrenergic signaling provides a safe and effective strategy for inhibiting multiple cellular, and molecular pathways related to metastasis and disease recurrence in early‐stage breast cancer.
Association of β‐blocker use at time of radical prostatectomy with rate of treatment for prostate cancer recurrence	[213]	Propanolol	NE	Use of nonselective β‐blockers was associated with less treatment initiation for cancer recurrence in prostate cancer.
Beta blockers and improved progression‐free survival in patients with advanced HER2 negative breast cancer: a retrospective analysis of the ROSE/TRIO‐012 study	[214]	β‐Adrenergic blocking drugs	NE	β‐Adrenergic blocking drugs intake was associated with significant improvement in PFS, particularly in patients with TNBC and patients not previously exposed to BB.
Propranolol attenuates surgical stress‐induced elevation of the regulatory T cell response in patients undergoing radical mastectomy	[215]	Propanolol	NE	Propranolol alleviates surgical stress‐induced elevation of Tregs in breast cancer patients.
Impact of beta blocker medication in patients with platinum sensitive recurrent ovarian cancer‐a combined analysis of 2 prospective multicenter trials by the AGO Study Group, NCIC‐CTG and EORTC‐GCG	[216]	Propanolol	NE	Response rates to chemotherapy, mPFS and mOS were not significantly different between patients treated with beta blockers and those who were not.
Beta‐blockers improve survival outcomes in patients with multiple myeloma: a retrospective evaluation	[217]	Propanolol	NE	β‐Blocker intake is associated with a reduced risk of disease‐specific death and overall mortality in patients with multiple myeloma.
Propranolol reduces cancer risk: a population‐based cohort study	[218]	Propanolol	NE	Propranolol can reduce the risk of head and neck, esophagus, stomach, colon, and prostate cancers.
Propranolol monotherapy in angiosarcoma—a window‐of‐opportunity study (PropAngio)	[219]	Propanolol	NE	This window‐of‐opportunity trial did not show clinical efficacy of propranolol monotherapy. However, two out of 14 patients did show clinical benefit. ADRB1/2 expression did not correlate with clinical response.
Pancreatic resection with perioperative drug repurposing of propranolol and etodolac—the phase II randomized controlled PROSPER trial	[220]	Propanolol	NE	Propranolol may help to improve short‐term postoperative immunity, reduce distance distant recurrence rate and increase DFS.
Phase II study of propranolol feasibility with neoadjuvant chemotherapy in patients with newly diagnosed breast cancer	[221]	Propanolol	NE	The propranolol–chemotherapy combination therapy demonstrated excellent tolerability in patients, with a mean propranolol dosing adherence rate of 96%. All enrolled patients successfully completed the treatment protocol and proceeded to scheduled surgical intervention.
Phase I clinical trial of combination propranolol and pembrolizumab in locally advanced and metastatic melanoma: safety, tolerability, and preliminary evidence of antitumor activity	[222]	Propanolol	NE	The propranolol–pembrolizumab combination therapy demonstrated a 78% objective response rate (ORR) in treated patients.
Prospective pilot trial with combination of propranolol with chemotherapy in patients with epithelial ovarian cancer and evaluation on circulating immune cell gene expression	[223]	β‐Adrenergic blocking drugs	NE	Sixty‐nine percent of patients (18/26) successfully completed the primary endpoint of six chemotherapy cycles while maintaining beta‐blocker therapy. The combination regimen significantly improved patient's outcomes, including overall quality of life (QoL), anxiety, and depression scores.
Phase 3 randomized, placebo‐controlled clinical trial of donepezil for treatment of cognitive impairment in breast cancer survivors following adjuvant chemotherapy (WF‐97116)	[224]	Donepezil	AchE	Donepezil did not improve memory problems caused by chemotherapy in breast cancer patients.
A study of donepezil in female breast cancer survivors with self‐reported cognitive dysfunction 1 to 5 years following adjuvant chemotherapy	[225]	Donepezil	AchE	The donepezil‐treated group demonstrated statistically significant improvements in two key memory parameters compared with controls: (1) HVLT‐R total recall and (2) HVLT‐R discrimination; but no significant differences on other cognitive variables or in subjective cognitive function or quality of life was observed.
A phase 0, window of opportunity study of parasympathetic stimulation with bethanechol in localized pancreatic adenocarcinoma prior to surgery	[226]	Bethanechol	Ach	R0 resections were achieved in nine patients (69%); there was no difference in Ki67 and CD44 tissue biomarkers between bethanechol‐treated and control samples. Decreased numbers of granzyme B‐expressing cells were seen in bethanechol‐treated tissues. Bethanechol treatment was associated with the suppression of circulating IL‐18.
Treatment of intractable cancer pain with resiniferatoxin—an interim study	[227]	Resiniferatoxin	TRPV1	Resiniferatoxin treatment was associated with decreased “worst” pain intensity by 38% and reduced opioid consumption by 57% measured at posttreatment day 15.
Standard pancreatoduodenectomy versus extended pancreatoduodenectomy with modified retroperitoneal nerve resection in patients with pancreatic head cancer: a multicenter randomized controlled trial	[228]	Retroperitoneal nerve resection		Retroperitoneal nerve resection demonstrated significant therapeutic benefits, including: (1) reduced locoregional and mesenteric lymph node recurrence rates (HR 0.52, 95% CI 0.31–0.87; *p* = 0.013), (2) improved disease‐free survival (median DFS 28.4 vs. 18.6 months; *p* = 0.007), and (3) sustained reduction in back pain at 6 months postoperatively.

**TABLE 3:** Ongoing clinical trials targeting the neuro–cancer crosstalk.

Study name	Trial number^a^	Drug	Targets	State
Propranolol hydrochloride in combination with sintilimab and platinum‐based chemotherapy for treatment of advanced non‐small cell lung cancer (BRIO)	NCT05979818	Propanolol	NE	Recruiting
The use of propranolol in the perioperative period of resectable gastrointestinal tumors	NCT06775080	Propanolol	NE	Recruiting
Immune checkpoint inhibitors with or without propranolol hydrochloride in patients with urothelial carcinoma	NCT04848519	Propanolol	NE	Recruiting
Perioperative use of a β‐adrenergic blocker, propranolol, and a COX2 inhibitor, etodolac, in patients undergoing resection with curative intent for primary colon and rectal cancer: effect on tumor recurrence and survival	NCT03919461	Propanolol	NE	Recruiting
Examining safety, efficacy and feasibility of preoperative propranolol in patients with PDAC (IMPULS)	NCT06145074	Propanolol	NE	Recruiting
Perioperative intervention to reduce metastatic processes in pancreatic cancer patients undergoing curative surgery (BC‐PC)	NCT03838029	Propanolol	NE	Recruiting
Durvalumab and tremelimumab in combination with propranolol and chemotherapy for treatment of advanced hepatopancreabiliary tumors (blocked)	NCT05451043	Propanolol	NE	Recruiting
A phase I study to evaluate the safety of naltrexone and propranolol in combination with standard of care ipilimumab and nivolumab in patients with advanced melanoma	NCT05968690	Propanolol naltrexone	NE opioid receptor	Recruiting
Propranolol with standard chemoradiation for esophageal adenocarcinoma a phase II study	NCT04682158	Propanolol	NE	Recruiting
Inhibiting beta‐adrenergic and COX‐2 signaling during the perioperative period to reduce ovarian cancer progression: a randomized placebo‐controlled clinical trial	NCT06839144	Propanolol	NE	Recruiting
An open label phase 2 study on propranolol and pembrolizumab in advanced angiosarcoma and undifferentiated pleomorphic sarcoma—a Scandinavian Sarcoma Group Collaboration	NCT05961761	Propanolol	NE	Recruiting
Propranolol in combination with pembrolizumab and standard chemotherapy for the treatment of unresectable locally advanced or metastatic esophageal or gastroesophageal junction adenocarcinoma	NCT05651594	Propanolol	NE	Recruiting
Propranolol and pembrolizumab for tumor re‐sensitization and treatment of patients with checkpoint inhibitor refractory metastatic or unresectable triple negative breast cancer	NCT05741164	Propanolol	NE	Recruiting
A study comparing perioperative stress reduction vs. standard of care in ovarian cancer (PRESERVE)	NCT05429970	Propanolol	NE	Recruiting
Nonchemotherapeutic interventions for the improvement of quality of life and immune function in patients with multiple myeloma	NCT05312255	Propanolol	NE	Recruiting
Phase II, neoadjuvant study of parasympathetic stimulation with bethanechol in combination with gemcitabine and nab‐paclitaxel in borderline resectable pancreatic adenocarcinoma	NCT05241249	Bethanechol	Ach	Recruiting
Resiniferatoxin to treat severe pain associated with advanced cancer	NCT00804154	Resiniferatoxin	TRPV1	Recruiting
Periganglionic resiniferatoxin for the treatment of intractable pain due to cancer‐induced bone pain	NCT02522611	Resiniferatoxin	TRPV1	Not yet recruiting

^a^ *Data sources*—clinical registration website (https://clinicaltrials.gov/).

However, developing neural‐targeted therapies that selectively disrupt tumor–nerve interactions while preserving normal neural function remains a significant challenge. Systemic application of these neural‐targeting agents at therapeutic doses required for antitumor efficacy may incur neurotoxic side effects, necessitating comprehensive evaluation of neurologic sequelae, including severity, duration, and reversibility, during therapeutic development. Additionally, a locally targeted drug delivery system using cancer‐targeted nanoparticles carrying agents targeting neural signaling represents a superior therapeutic strategy for minimizing systemic unintended off‐target effects associated with systemic administration of neural‐modulating agents. As interventional physicians, we recognize intra‐arterial catheter‐directed delivery of neural‐modulating agents as a promising avenue for the clinical translation of neuro‐targeted therapy for cancer treatment. Moreover, endovascular denervation may be a promising approach for the clinical translation of neuro‐targeted therapy for cancer. Advances in neurovascular anatomy revealed the intimate spatial relationship between peripheral nerves and arterial networks, prompting investigation into intra‐arterial catheter‐based ablation systems for targeted neural disruption [229]. Endovascular denervation technology originated with the pioneering work of Professor Krum in 2009, who developed the first transcatheter renal denervation system [230]. This innovative approach may facilitate precise modulation of tumor‐associated nerve signaling while minimizing systemic effects. The intrahepatic sympathetic neural network originates from the celiac plexus, coursing along the hepatic artery and its branches. These perivascular nerves penetrate the liver parenchyma and biliary system, forming the anatomical foundation for transvascular ablation denervation techniques. Hepatic sympathetic innervation patterns provide a rationale for targeted neuromodulation in hepatic disorders [162, 231, 232]. We are conducting a clinical trial to evaluate the therapeutic efficacy of ablating sympathetic nerves via intravascular radiofrequency in patients with hepatocellular carcinoma (NCT06694636), aiming to achieve encouraging results and pave the way for the use of sympathetic nerve inhibition in treating the disease.

Neural‐targeted therapies can low tumor progression, however, complete tumor regression is difficult to achieve using monotherapies. Accumulating evidence demonstrates that neural signaling modulates tumor cell sensitivity to conventional treatments, including radiation, chemotherapy, and immunotherapy, thereby promoting therapeutic resistance. Propranolol, a selective β‐blocker, can be used as an adjunctive therapy in cancer treatment, particularly in reducing the risk of recurrence during the perioperative period [233]. Clinical studies demonstrate that β‐adrenergic receptor blockade synergizes with chemotherapy to delay hepatic metastasis and improve patient outcomes significantly [191]. In addition to chemotherapy, β‐blockers exert synergistic antitumor effects with immune checkpoint inhibitors in patients with non‐small‐cell lung cancer, improving their prognosis [234]. Further clinical validation is warranted for the use of nervous signaling intervention as an adjuvant therapy for conventional antitumor therapy.

The translation of the concept of neural regulation of cancer into clinical practice remains a significant challenge. On the one hand, evidence confirming the regulatory role of nerves in tumor progression remains limited, and conclusions drawn from existing preclinical research models may not be entirely reliable. For instance, different studies have yielded conflicting results on whether the parasympathetic nerve inhibits or promotes pancreatic cancer progression. This inconsistency may be related to variations in the cell lines and animal models used. On the other hand, the regulatory effect of the nervous system are highly complex, as evidenced by the fact that different types of neural signals can exert either synergistic or antagonistic effects on the same target cell. Neuro–immune–tumor interactions may exhibit different functions and effects under different conditions. The TME is composed of diverse cellular components, all of which may be modulated by neural signals, exacerbating the complexity of neural regulation in tumors. However, most studies focus on investigating the impact of a single neural signal on a specific cell type using animal models, which may not accurately recapitulate the intricate neural–immune–tumor regulatory network present in human cancer tissues. The use of precise molecular biology tools is required for unraveling the mechanisms underlying the neural–immune–tumor regulatory network. The advancement of spatial and single‐cell omics technologies will facilitate research in tumor neurobiology [235]. However, the vast and intricate datasets generated by these experimental methods‐based cancer neuroscience research require advanced bioinformatics tools and statistical approaches for effective processing and meaningful interpretation. Furthermore, neural signaling plays a crucial regulatory role in the function of normal tissues, and targeting these pathways for tumor suppression without affecting their physiological functions represents a significant therapeutic challenge. Systemic administration of cancer‐targeted nanoparticle carrying agents targeting neural signaling and locally ablating nerves via intravascular radiofrequency might represent alternative options.

Despite the numerous challenges in the field of neuro–immune–cancer interaction research, the development of precise molecular biology tools and techniques could help overcome these challenges. This research direction holds tremendous potential for developing novel anticancer therapeutic strategies, and further efforts are required to promote cancer neuroscience research.

## Author Contributions

Gao‐Jun Teng and Hai‐Dong Zhu conceived the manuscript. Yan Fu, Zhi‐Shan Ge, and Qing‐Yue Cao reviewed the literature, drafted the manuscript, and drew the figures. Gao‐Jun Teng supervised and revised the manuscript. Yan Fu, Zi‐Han Li, and An Zhang revised the manuscript according to the editor's and reviewers’ suggestion. All authors read and approved the final manuscript.

## Funding

This work was supported by Natural Science Foundation of Jiangsu Province (No. BK20241693), China Postdoctoral Science Foundation (No. 2024M750449), National Natural Science Foundation of China (82072039, 82372067), National Key R&D Program of China (No. 2023YFC2413500), and Collaborative Innovation Center of Radiological Medicine of Jiangsu Higher Education Institutions (FY202302).

## Ethics Statement

The authors have nothing to report.

## Conflicts of Interest

The authors declare no conflicts of interest.

## Data Availability

The data that support the findings of this study are available in the paper.
